# ChatGPT yields low accuracy in determining LI-RADS scores based on free-text and structured radiology reports in German language

**DOI:** 10.3389/fradi.2024.1390774

**Published:** 2024-07-05

**Authors:** Philipp Fervers, Robert Hahnfeldt, Jonathan Kottlors, Anton Wagner, David Maintz, Daniel Pinto dos Santos, Simon Lennartz, Thorsten Persigehl

**Affiliations:** ^1^ Department of Diagnostic and Interventional Radiology, University Cologne, Faculty of Medicine and University Hospital Cologne, Cologne, Germany; ^2^ Department of Diagnostic and Interventional Radiology, Goethe University Frankfurt am Main, University Hospital Frankfurt, Frankfurt am Main, Germany

**Keywords:** diagnostic imaging, neoplasms, liver, diagnosis, LI-RADS (liver imaging reporting and data system), MRI

## Abstract

**Background:**

To investigate the feasibility of the large language model (LLM) ChatGPT for classifying liver lesions according to the Liver Imaging Reporting and Data System (LI-RADS) based on MRI reports, and to compare classification performance on structured vs. unstructured reports.

**Methods:**

LI-RADS classifiable liver lesions were included from German written structured and unstructured MRI reports with report of size, location, and arterial phase contrast enhancement as minimum inclusion requirements. The findings sections of the reports were propagated to ChatGPT (GPT-3.5), which was instructed to determine LI-RADS scores for each classifiable liver lesion. Ground truth was established by two radiologists in consensus. Agreement between ground truth and ChatGPT was assessed with Cohen's kappa. Test-retest reliability was assessed by passing a subset of *n* = 50 lesions five times to ChatGPT, using the intraclass correlation coefficient (ICC).

**Results:**

205 MRIs from 150 patients were included. The accuracy of ChatGPT at determining LI-RADS categories was poor (53% and 44% on unstructured and structured reports). The agreement to the ground truth was higher (*k* = 0.51 and *k* = 0.44), the mean absolute error in LI-RADS scores was lower (0.5 ± 0.5 vs. 0.6 ± 0.7, *p* < 0.05), and the test-retest reliability was higher (ICC = 0.81 vs. 0.50), in free-text compared to structured reports, respectively, although structured reports comprised the minimum required imaging features significantly more frequently (Chi-square test, *p* < 0.05).

**Conclusions:**

ChatGPT attained only low accuracy when asked to determine LI-RADS scores from liver imaging reports. The superior accuracy and consistency throughout free-text reports might relate to ChatGPT's training process.

**Clinical relevance statement:**

Our study indicates both the necessity of optimization of LLMs for structured clinical data input and the potential of LLMs for creating machine-readable labels based on large free-text radiological databases.

## Highlights

• ChatGPT (GPT-3.5) was not capable of accurately classifying liver MRI reports according to LI-RADS.
• Only 53% and 44% of *n* = 428 liver lesions were classified correctly for free-text and structured reports, respectively.
• Classification based on German written free-text reports resulted in higher agreement with ground truth diagnoses, higher consistency, and lower mean absolute error in LI-RADS scores.

## Background

Today, a lack of high-quality annotated data is still one of the greatest hurdles for the development of artificial intelligence (AI) applications in modern medicine (1). Training of artificial neural networks for medical purposes requires large amounts of annotated data, which are usually obtained through the bottleneck of manual data labelling (2). Recent efforts in medical imaging have aimed to develop machine-readable structured reports, to facilitate the process of data labelling (3–5). Such structured radiology reports have further proven to be more consistent and comprehensive than free-text reports (3, 4, 6). In line with these investigations, several radiological societies advocate for the adoption and use of structured reporting (7–9). Nonetheless, it is still common practice that radiology reports are written in a free-text (i.e., unstructured) manner, which hampers automated data mining and introduces discrepancies of interpretation between radiologists and clinicians (10).

A blueprint for a contemporary structured reporting framework is the Liver Imaging Reporting and Data System (LI-RADS) (11). LI-RADS classifies primary liver tumors of high-risk patients into five categories, assessing the risk of malignancy of the described liver lesion. The LI-RADS category of a liver lesion, and hence its probability of malignancy, depends on imaging features such as its size and contrast enhancement dynamics. If the required imaging features are comprehensively documented, the LI-RADS algorithm can be used to determine a distinct, unambiguous category from LR-1 to LR-5 (11). Such highly structured reports of liver imaging support automated data mining and hence enable training of neural networks without time consuming manual data curation of free-text reports (11, 12).

In the last decade, AI-based natural language processing (NLP) networks have been suggested as an option to support interpretation and data mining in the field of radiology; however, performance strongly depends on training data size (13). Recently, large language models (LLMs) have been in the spotlight of scientific and public attention. One of the most recent LLMs is ChatGPT (14). Developed by OpenAI (San Francisco, CA), ChatGPT was trained using a large amount of text data from several decades with over 175 billion parameters (14, 15). By utilizing deep neural networks, ChatGPT generates responses in natural language based on text inputs through the form of a chat prompt (15). ChatGPT has already proven convenient in several use cases, such as customer support, e-commerce, education, and evaluation of medical inquiries (15, 16).

The hypothesis of this study was that LLMs like ChatGPT might enable classification of lesions based on imaging findings, thereby holding the potential for automated transformation of radiology reports into structured data labels. Due to its unambiguous character and distinct terminology of imaging findings, the LI-RADS scale has been suggested as a promising framework for such NLP pilot studies (17). In this study, we therefore investigated if ChatGPT is capable of transforming radiology MRI reports of liver lesions into LI-RADS classifications. Further, we assessed possible differences in classification performance when processing structured and unstructured reports.

## Methods

This single-center study was performed in accordance with the ethical standards of the institutional (application number 23-1061-retro) and national research committee, and with the 1964 Helsinki declaration and its later amendments or comparable ethical standards. Informed consent was waived due to retrospective study characteristics. This study was conducted without any violation of ethical standards or legal frameworks. All reports based on MRI examinations conducted at our clinic (Radiology department of the University Hospital Cologne).

### Patient enrollment

Reports and corresponding liver lesions were included by reviewing the institutional database for the following eligibility criteria:

1. Magnetic resonance imaging (MRI) examination between January 1st 2010 and January 1st 2022, according to the LI-RADS technical recommendations (11).
2. High-risk population for hepatocellular carcinoma (HCC), according to the LI-RADS definition (11).

Our in-house database comprises unstructured, free-text reports and structured reports, which are composed according to a reporting template. The reporting template requires specific imaging findings to be described (e.g., contrast dynamics of liver lesions), yet leaves some degree of freedom to the radiologist in wording, editing, and arrangement of findings. In clinical practice, those structured reports might hence include sections of unstructured free-text. Our database comprises reports written in German language.

From all eligible reports, we initially selected a random subsample of *n* = 250 unstructured and *n* = 250 structured reports of LI-RADS classifiable, non-treated liver lesions. Consecutively, the reports were screened for the quality of documentation by a blinded radiologist with 5 years of experience in liver imaging. Reports with insufficient quality information to support an unambiguous LI-RADS classification were excluded: this applied for lesions without documentation of at least lesion size and enhancement in the arterial contrast phase. Documentation of lesion location (liver segments reported) was another mandatory requirement to allow for unambiguous identification of the lesion. If a major feature of the LI-RADS classification was not reported (e.g., washout in delayed venous phase), we assumed that it was not present. Finally, 178 lesions from unstructured and 250 from structured reports were included for the final analysis.

### Imaging protocol

Liver MRI was performed according to the LI-RADS technical recommendations (11). Examinations included unenhanced T1-weighted imaging, T2-weighted imaging, T2-weighted imaging with fat suppression, diffusion weighted imaging, as well as multiphase contrast-enhanced T1-weighted FS imaging (precontrast, arterial, portal venous, and delayed phases).

### Assessment of the ground truth LI-RADS category

The ground truth LI-RADS category was determined by two experienced radiologists in liver imaging in a consensus reading based on the radiology reports (5 and more than 15 years of experience in liver imaging).

### Assessment of reports by ChatGPT

All communication between us and ChatGPT was conducted in German. The “findings” section of the radiology report was transferred to ChatGPT (GPT-3.5, OpenAI, CA, USA) one-by-one by an independent radiologist, blinded to the ground truth LI-RADS category, without specific user interaction. If the LI-RADS classification of a liver lesion was mentioned in the “findings” section of the report, this statement was removed to assure unbiased comparability of the included lesions. The reports were not abbreviated or rearranged; hence including incidental findings, description of non-liver findings, and possible typographical errors (typos). To avoid bias by context sensitivity of ChatGPT in the course of the analysis, the chat prompt was restarted before each inquiry. Together with the radiology report, a short description of the task was further input to ChatGPT (Please note that the following text was entered to ChatGPT in German language and has been translated into English as part of this paper for better understanding):

*Can you classify the following MRI report according to the LI-RADS classification structured by lesion and liver segment? If a feature is not mentioned in the report (e.g., venous washout), it is not present. Please mention only the lesions that can be classified according to LI-RADS and answer in bullet points according to the following scheme: Lesion Nr.: XX; Size: XX, Segment: XX, LI-RADS: XX*

**Figure 1:**
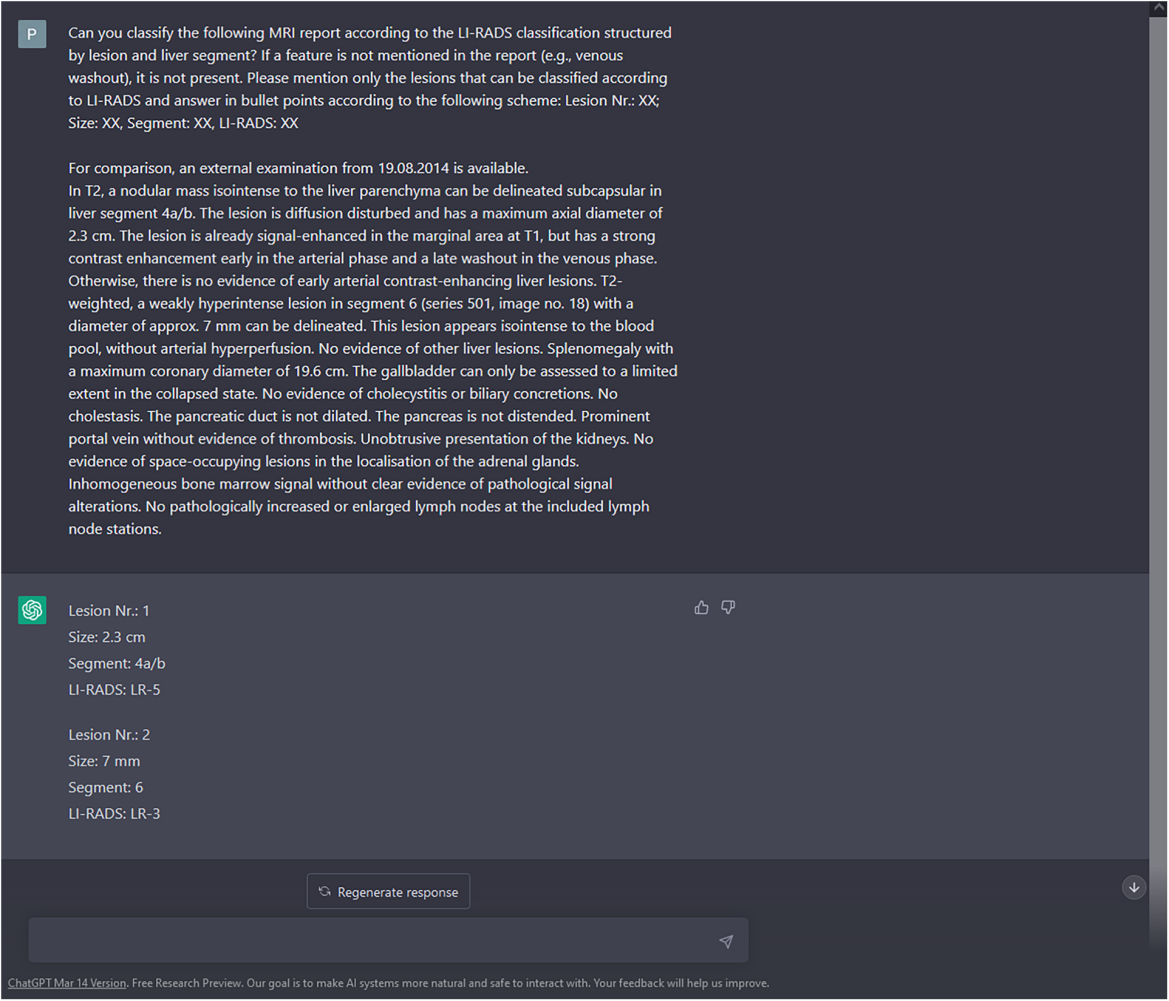
Enrollment of liver lesions. Along with the query to create a structured LI-RADS imaging report, the findings section of the MRI report was copied to the ChatGPT prompt without specific user interaction (https://chat.openai.com/chat). Besides description of liver lesions, possible incidental findings and non-liver pathologies were included. In this exemplary case, both liver lesions were correctly classified by ChatGPT according to the ground truth of two experienced radiologists. To preclude interference with ChatGPT's context sensitivity, the prompt was restarted after each query. Please note that for reasons of understandability, the report was translated from German to English prior to this query. In the present study, MRI reports were processed by ChatGPT without prior translation.

The LI-RADS category suggested by ChatGPT was then matched to the ground truth LI-RADS category, based on the lesion's size and location. An exemplary query of ChatGPT is shown in Figure 1.

### Statistical data assessment

Statistical analysis was performed in *R* language for statistical computing, R Foundation, Vienna, Austria, version 4.0.0 (18). Visualization was done using the R library ggplot2 (19). A *p*-value <0.05 was considered statistically significant. Agreement of ground truth and ChatGPT's ratings was assessed by weighted Cohen's kappa k for two raters, calculated by using the R library *irr* (20). The test-retest reliability was assessed on a subset of *n* = 25 lesions reported in free-text and *n* = 25 lesions reported in a structured manner, while each lesion was rated five times by ChatGPT (total additional number of lesion ratings for test-retest analysis *n* = 250). We calculated the test-retest reliability of ChatGPT's ordinally scaled LI-RADS ratings by intraclass correlation coefficients (ICC) in a single-rater, absolute agreement model using the R library *psych* (21, 22)*.* Power analysis was performed *post hoc* by G*power, since the difference of performance between unstructured and structured reports could not be estimated *a priori*, due to a lack of comparable studies (23).

## Results

**Figure 2:**
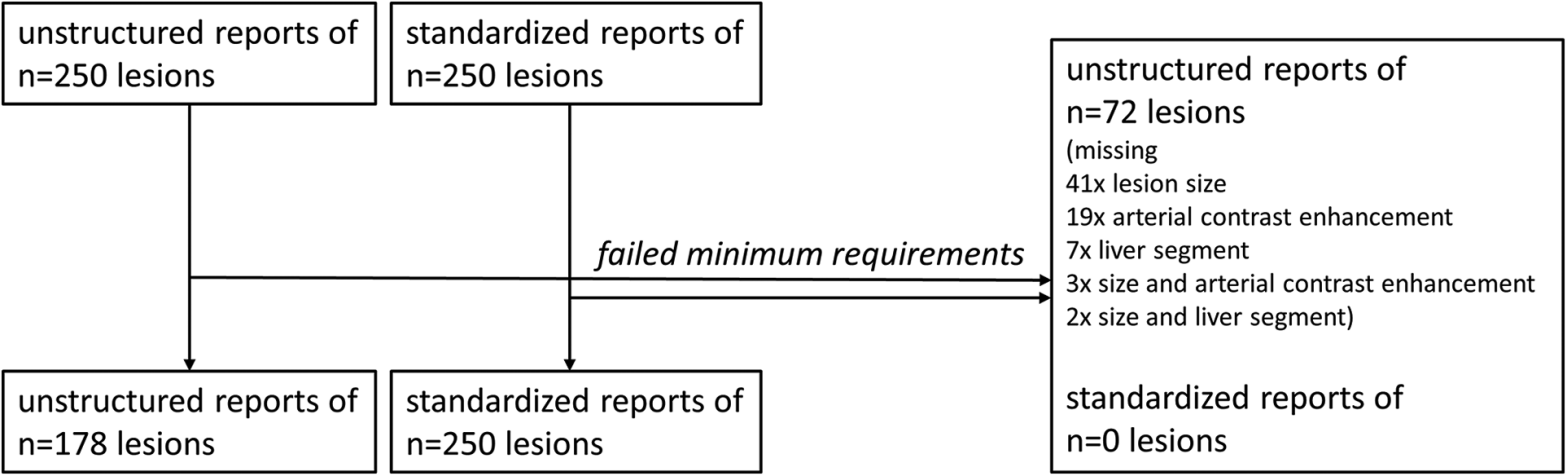
Enrollment of liver lesions.

In total, we analyzed *n* = 428 liver lesions after exclusion of *n* = 72 lesions. Liver lesions from unstructured reports missed the minimum required imaging features, i.e., lesion size, location, and arterial contrast dynamics, significantly more often (Chi-square test, *p* < 0.05). All liver lesions documented in structured reports met the minimum requirements for inclusion. Figure 2 illustrates the enrollment of liver lesions.

**Figure 3:**
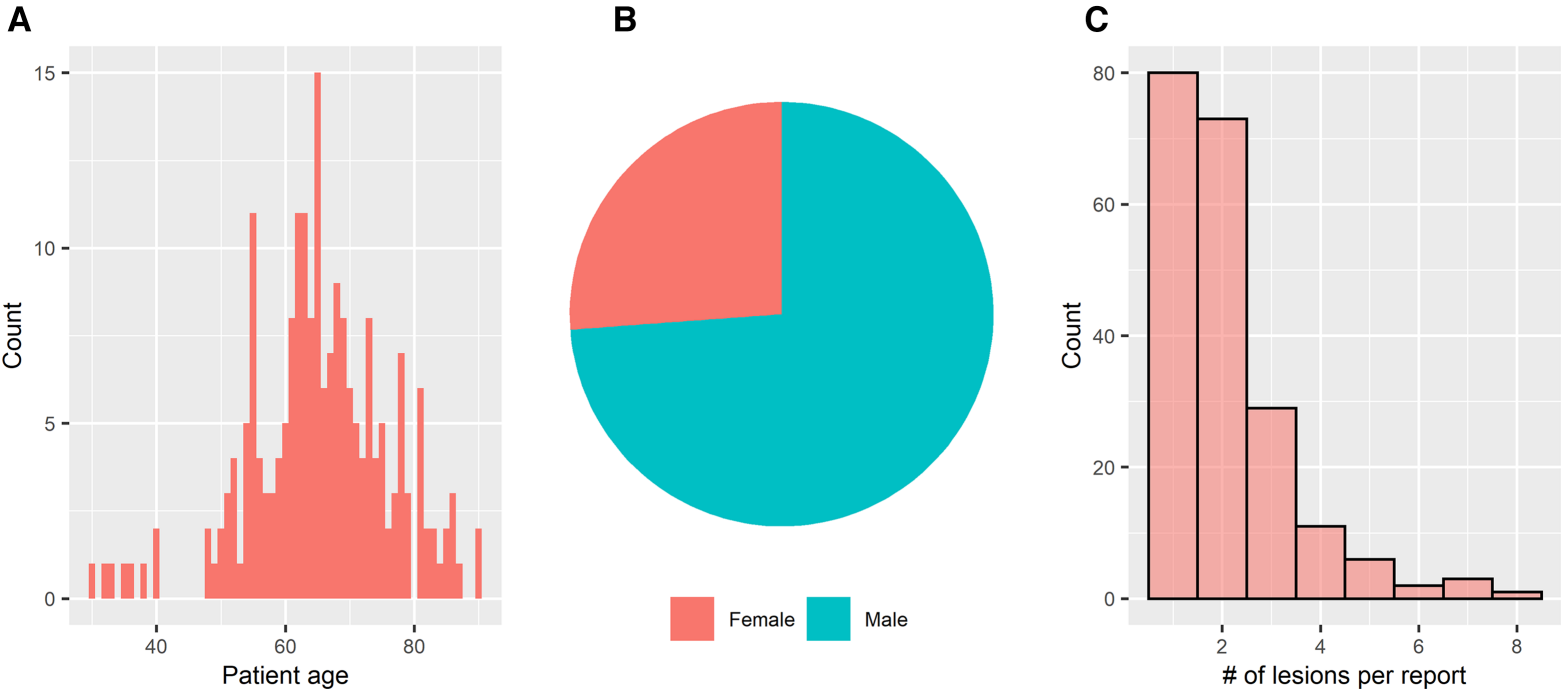
Basic statistics. Mean age of the analyzed patient population was 65.5 ± 10.9 years (**A**) 74% (*n* = 111) of the included high-risk patients were male (**B**). The median number of included lesions per radiology report was 2 [1–3], with the most common categories of 1 (80/205 reports, 39%), 2 (73/205 reports, 36%), and 3 (29/205 reports, 14%) included lesions per report, respectively (**C**).

After exclusion of liver lesions that did not meet the minimum requirements, *n* = 205 radiology reports from *n* = 150 high-risk patients were analyzed. The mean number of included lesions per report was 2.1 ± 1.3. Mean age of the analyzed patient population was 65.5 ± 10.9 years. 74% (*n* = 111) of the included high-risk patients were male. The median number of included lesions per radiology report was 2 [1–3], with 89% (182/205 reports) including ≤3 lesions. Basic descriptive statistics are summarized in Figure 3.

### Descriptive statistics of the ground truth and ChatGPT-based LI-RADS ratings

The mean ground truth LI-RADS categories were 4.4 ± 0.8 and 3.5 ± 0.9 for unstructured and structured reported liver lesions, respectively. In four patients, LI-RADS 1 lesions were reported as the ground truth. In 7, 82, 46, and 88 patients, LI-RADS 2–5 lesions were reported as the ground truth, respectively. Since more than one report per patient could be included to our study, the sum surpasses the total number of patients. The median number of reports per patient was 1 [1–2] in our study. The highest LI-RADS category per patient was 1 for one patient, 2 for three patients, 3 for 38 patients, 4 for 20 patients, and 5 for 88 patients, respectively. Ground truth LI-RADS classifications were significantly higher throughout unstructured reports (Wilcoxon test, *p* < 0.05). ChatGPT classified 16% (*n* = 70) and 3% (*n* = 11) of liver lesions as LR-4a and LR-4b, respectively. Since LR-4a and LR-4b are no valid categories according to the LI-RADS manual, these lesions were adopted as LR-4 in the following analysis. ChatGPT-based LI-RADS ratings were 4.2 ± 0.6 and 3.7 ± 0.8 for unstructured and structured reported liver lesions. ChatGPT ratings were significantly lower than the ground truth throughout unstructured reports, and significantly higher throughout structured reports (Wilcoxon test, *p* = 0.01 and *p* < 0.001, respectively). The frequency distribution of LI-RADS ratings is reported in detail in Table 1.

**Table 1:** Frequency distribution of LI-RADS ratings.

LI-RADS category	Unstructured reports		Structured reports	
	Ground truth	ChatGPT	Ground truth	ChatGPT
1	0	0	2% (5/250)	1% (3/250)
2	0	1% (2/178)	4% (10/250)	8% (20/250)
3	21% (37/178)	7% (13/178)	57% (144/250)	21% (53/250)
4	21% (37/178)	57% (102/178)	18% (44/250)	60% (150/250)
5	58% (104/178)	34% (61/178)	19% (47/250)	10% (24/250)

LI-RADS ratings were significantly higher throughout unstructured reports compared to the structured reports, which applied for the ground truth as well as ChatGPT's ratings (Wilcoxon test, *p* < 0.05).

### Assessment of ChatGPT's classification performance

**Figure 4:**
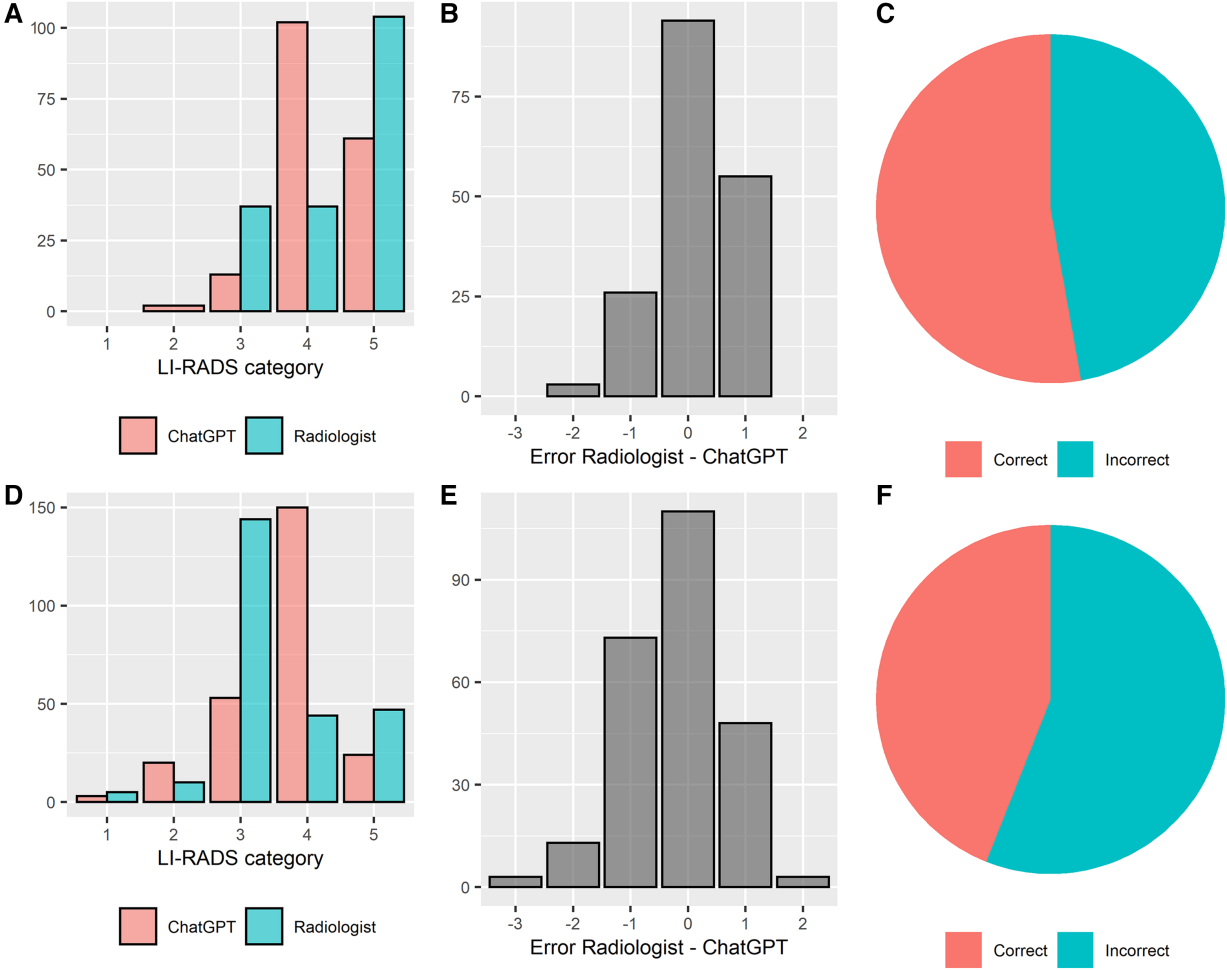
LI-RADS classification performance of ChatGPT based on unstructured and structured radiology reports. Performance overview of unstructured and structured reports is shown in the top (**A**–**C**) and bottom row (**D**–**F**), respectively. (**A**/**D**) distribution of the LI-RADS scores, (**B**/**E**) errors between the experienced liver radiologist and ChatGPT, (**C**/**F**) percentage of correct and incorrect LI-RADS classifications by ChatGPT.

ChatGPT correctly classified 53% (94/178) and 44% (110/250) of liver lesions reported in an unstructured and structured manner, respectively. There was a tendency that unstructured reports were classified correctly more often, however, without attaining statistical significancy (Chi-square test, *p* = 0.07). The agreement between ground truth and ChatGPT's LI-RADS ratings was moderate, with a weighted Cohen's kappa of *k* = 0.51 and *k* = 0.44 for unstructured and structured reports, respectively. Median absolute error of the ChatGPT-based ratings compared to the ground truth was 0.5 ± 0.5 LI-RADS categories in the unstructured reports and 0.6 ± 0.7 LI-RADS categories in the structured reports, respectively. In 96% (81/84) and 86% (121/140) of incorrectly rated lesions, ChatGPT's rating was wrong by only 1 LI-RADS category, for unstructured and structured reports, respectively. The absolute error was significantly lower throughout unstructured reports (Wilcoxon test, *p* < 0.05). The performance of LI-RADS classifications by ChatGPT is illustrated in Figure 4.

To investigate if there was a systematic error of ChatGPT's ratings, Table 2 lists the direction of error along with its magnitude for lesions in each ground truth category.

**Table 2:** In-detail report of ChatGPT's error for lesions grouped by ground truth categories.

Ground truth	Structured reports		Unstructured reports	
	ChatGPT	Mean error	ChatGPT	Mean error
LI-RADS 1	3.0 ± 1.4	+2.0 ± 1.4	NA	
LI-RADS 2	3.3 ± 0.9	+1.3 ± 0.9	NA	
LI-RADS 3	3.5 ± 0.8	+0.5 ± 0.8	3.6 ± 0.7	+0.6 ± 0.7
LI-RADS 4	3.9 ± 0.4	−0.1 ± 0.4	4.2 ± 0.4	+0.2 ± 0.4
LI-RADS 5	4.4 ± 0.5	−0.6 ± 0.5	4.5 ± 0.5	−0.5 ± 0.5

The direction of ChatGPT's error is outlined by +/– ahead of the mean error's magnitude (e.g., mean error +2.0 means that ChatGPT overestimated lesions in the respective category by 2.0 LI-RADS categories).

The largest magnitude of ChatGPT's error was observed in ground truth LI-RADS 1, 2, and 3 lesions (overestimation by a mean of 2.0, 1.3, and 0.5 LI-RADS categories in structured reports, respectively). The minimum classification error was observed in ground truth LI-RADS 4 lesions (mean error of −0.1 and +0.2 LI-RADS categories for unstructured and structured reports, respectively). Ground truth LI-RADS 5 lesions were underestimated by a mean of −0.6 and −0.5 LI-RADS categories.

### *Post hoc* power analysis to validate the sample size

The Chi square test to assess the difference of performance in free-text and unstructured reports yielded a statistical power of *β *= 0.96, which surpassed the desired power level of 0.80. The power analysis is reported in detail in Supplementary Material S1.

### Assessment of ChatGPT's test-retest reliability

In the test-retest reliability analysis, among the 25 free-text and 25 structured reports, 60% (15/25) and 24% (6/25) of liver lesions were consistently rated the identical LI-RADS score in all 5 iterations, respectively (significantly higher in free-text reports, Chi-square test, *p* < 0.05). The ICC was 0.81 and 0.50 for free-text and unstructured reports, which corresponds to a “good” and “moderate” test-retest reliability, respectively.

## Discussion

The present study investigated automated label generation in the form of LI-RADS categories, using ChatGPT on a sample of 428 liver lesions reported in free-text and structured MRI reports. ChatGPT correctly classified 53% and 44% of liver lesions in unstructured and structured reports on the LI-RADS scale, respectively. The agreement between ground truth and ChatGPT's ratings was overall moderate, yet superior throughout unstructured reports (weighted Cohen's kappa *k* = 0.51 and *k* = 0.44 for unstructured and structured reports, respectively). Mean error of ChatGPT's ratings compared to the ground truth was 0.5 ± 0.5 and 0.6 ± 0.7 LI-RADS categories throughout unstructured and structured reports, respectively. Although ChatGPT missed the correct LI-RADS rating in most cases by only 1 category, the poor accuracy indicates that ChatGPT currently it is not feasible to use for automated label generation on the LI-RADS scoring system.

ChatGPT's ratings showed a tendency towards the LI-RADS 4 category, with the largest error at the lower margin of the LI-RADS spectrum. In cognitive science, the trend of human judgements away from the extreme ends of a scale towards more moderate ratings is called the central tendency bias (24). Since other typical human cognitive biases have been observed in the interaction with LLMs, e.g., the framing effect, anchoring bias, or availability bias, the central tendency bias might be one possible explanation for this finding (25).

Identification of relevant imaging features from a radiology report and application the LI-RADS flowchart requires in-depth understanding of the LI-RADS definition. Other authors have proposed that ChatGPT lacks comprehensive knowledge of scientific literature and produces false or misleading text when detailed literature knowledge is required (26). This finding is in line with the poor accuracy of ChatGPT's LI-RADS ratings in our study. A crucial lack of “expert knowledge” can further be assumed due to the 19% (*n* = 81) of liver lesions that ChatGPT classified as LR-4a or LR-4b—which are categories that do not exist in the LI-RADS manual (11). The observations of non-existent LI-RADS categories are consistent with the phenomena of artificial hallucination (27). ChatGPT is known to be prone to artificial hallucination, confidently providing incorrect answers that are not covered by its training data (27).

As an unexpected result, we observed a superior classification accuracy and consistency of ChatGPT's ratings on unstructured reports vs. structured reports, albeit structured reports contained all relevant imaging features more often. In our study, the structured reports contained a broader spectrum of liver lesions, farther extending to the lower end of the LI-RADS scale. This agrees with the frequent observation, that structured reports tend to be more comprehensive compared to free-text reports (3). To elaborate on the effect of the different distribution of LI-RADS scores in the free-text and structured reports, and a possibly introduced bias to the accuracy analysis, we repeated the accuracy analysis on an artificially balanced dataset (Supplementary Material S2). Yet, within the balanced dataset, we again observed that ChatGPT yielded significantly more correct LI-RADS ratings based on the free-text reports. Hence, the superior accuracy of ChatGPT on free-text reports is not explainable by the different distribution of ground truth LI-RADS scores alone. Another possible explanation the above-mentioned finding might be an effect of the training process of the LLM: the chatbot was trained with human conversations and learned the statistical associations of words during the training process, rather than “understanding” their meanings (26, 28). A scarcity of words, such as a telegram style structured radiology report, might impede ChatGPT from concluding the correct statistical associations. The traditional way of dictating radiology reports in a stream-of-consciousness manner resembles human conversation, and hence ChatGPT's training dataset, more closely (3). On the other hand, the inferior quality of the unstructured radiology reports did not seem to influence the accuracy of ChatGPT's ratings, which brings up the question, if ChatGPT actually identified the relevant imaging features and applied the LI-RADS algorithm. In line with our data, similarly poor results have been observed when asking ChatGPT to deliver correct answers to bullet-point-like mathematical questions (29).

In the past years, non-LLM NLP models have already been investigated as a means to improve reporting and data processing in radiology. Yim et al. investigated a specifically trained NLP for extraction of HCC tumor information in 101 radiology reports (30). They focused on clinically relevant tumor staging information, including the number of HCC lesions, size, and anatomical location. Based on the annotated cohort, their label extraction system achieved an excellent labelling accuracy of the investigated items. This specifically trained NLP achieved a superior performance of liver specific labeling compared to our ChatGPT LI-RADS ratings, although they were based on very limited training data compared to ChatGPT. However, it is important to mention that previous studies using NLP for data labelling in radiology did not include the classification task, i.e., the automated suggestion of a LI-RADS category based on certain imaging findings.

A major limitation of our study is introduced by the untransparent way of data processing by ChatGPT. The chatbot is designed as a black box, only revealing its output text. Thus, it remains unclear if ChatGPT applied the LI-RADS algorithm, or if the LI-RADS ratings resulted from statistical associations of suggestive wordings, that were learned during the training process. After all, the purely statistical approach by ChatGPT might closely resemble the human conception of following the LI-RADS classification flow chart. Yet, the exact content of ChatGPT's training dataset is not disclosed, which introduces the question if it has ever been trained on the LI-RADS algorithm at all (31). Further, we did not use the most recent version GPT4 to perform the LI-RADS classifications. Dedicated studies are required to explore the possible benefit when scoring LI-RADS classifications by GPT4, compared to GPT3.5. Another minor limitation is the retrospective character of our study—yet, it resembles the use case of automated retrospective data labelling. Since the present study evaluated reports written in German, the evidence concerning other languages is limited; however, we consider the limitation of language only minor, since ChatGPT has proven excellent capabilities to process text without limitation of language (32). Further dedicated studies might investigate a possible benefit of prior translation to English language when processing the report by ChatGPT. Further, we did not perform a detailed analysis if ChatGPT's classification accuracy depends on the report-writing radiologist's experience. Throughout unstructured reports, the individual variation in presentation and wording of the reports might affect ChatGPT's performance. Since the presented study is monocentric, the characteristics of our in-house dataset might preclude generalization of the results.

## Conclusion

ChatGPT was not capable of yielding accurate LI-RADS ratings from liver imaging reports concerning our in-house dataset, yet performed better on unstructured data. Our results indicate both the necessity of optimization of LLMs for structured data input as well as the potential of LLMs for creating machine-readable labels based on large free-text radiological databases.

## Data Availability

The original contributions presented in the study are included in the article/**Supplementary Material**, further inquiries can be directed to the corresponding author.
