# Supplementary material for: ChatGPT yields low accuracy in determining LI-RADS scores based on free-text and structured radiology reports in German language
Source: Front Radiol. 2024 Jul 5;4:1390774. doi: 10.3389/fradi.2024.1390774 (PMC11257913; doi:10.3389/fradi.2024.1390774)
Supplement: Supplementary file 1 [file Datasheet1.docx]

**Supplementary material 1: Power analysis.**

The present study had two main objectives, as stated in the Abstract and Introduction:
1) To investigate the accuracy of ChatGPT in classifying liver lesions according to LI-RADS based on MRI reports, and
2) to assess possible differences in classification performance throughout structured and unstructured reports.

The first objective was answered by the absolute numbers of correctly classified lesions (53% and 44%), which did not require any statistical tests and, hence, there was no need for power analysis.

To answer the second objective, we performed a Chi square test of correctly classified liver lesions in unstructured vs. structured reports. The power analysis by G*power is described in detail below (Figure 1).


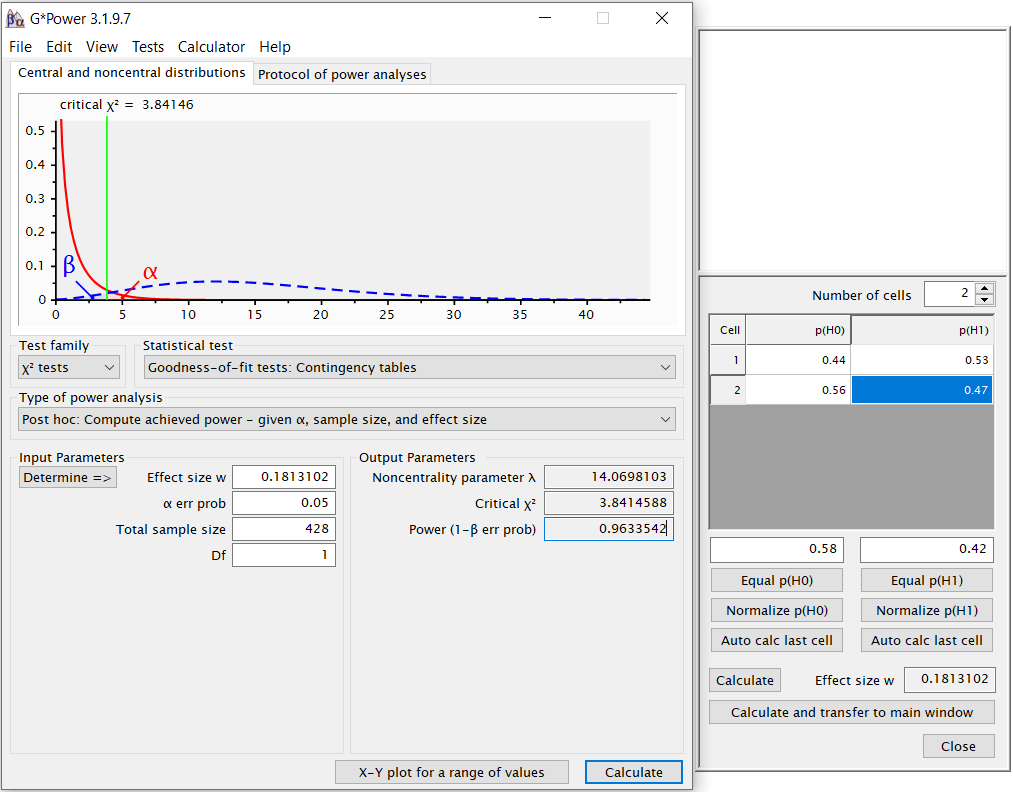


Figure 1: Post hoc power analysis in G*power (Chi square test, goodness-of-fit).
The input parameters were determined as follows:
**Effect size w**: calculated in the smaller window on the right, which shows the contingency table of the Chi square test. Relative frequencies of correctly vs. incorrectly classified lesions (top vs. bottom row), in unstructured vs. structured reports (left vs. right column), respectively. Below, the distribution of unstructured vs. structured reports is noted (0.58 vs. 0.42). The effect size w =0.18 is automatically calculated and transferred to the left side.
**Alpha error prob**: 0.05 (significance level)
**Total sample size**: 428
**Df (degrees of freedom)**: 1 (2 possible outcomes -1)

The output notes β =0.96, which exceeds the typically desired power level of 0.80.
