# Supplementary material for: ChatGPT yields low accuracy in determining LI-RADS scores based on free-text and structured radiology reports in German language
Source: Front Radiol. 2024 Jul 5;4:1390774. doi: 10.3389/fradi.2024.1390774 (PMC11257913; doi:10.3389/fradi.2024.1390774)
Supplement: Supplementary file 2 [file Datasheet2.docx]

**Supplementary material 2: Accuracy analysis in a balanced dataset**

The structured reports in our study contained relatively more lesions that were classified as ground truth LI-RADS 1 and 2. The different distribution of ground truth LI-RADS scores between unstructured and structured reports might bias the comparative analysis. Hence, this supplementary material repeated the analysis on an artificially balanced dataset. For each included lesion in a specific LI-RADS category from the unstructured or structured dataset, a corresponding lesion from the respective other dataset was randomly included. The two artificially balanced datasets each contained 37, 37, and 47 lesions in ground truth LI-RADS categories 3, 4, and 5, respectively. ChatGPT’s performance on these datasets is illustrated below in Figure 1, analogously to Figure 3 of the main manuscript.


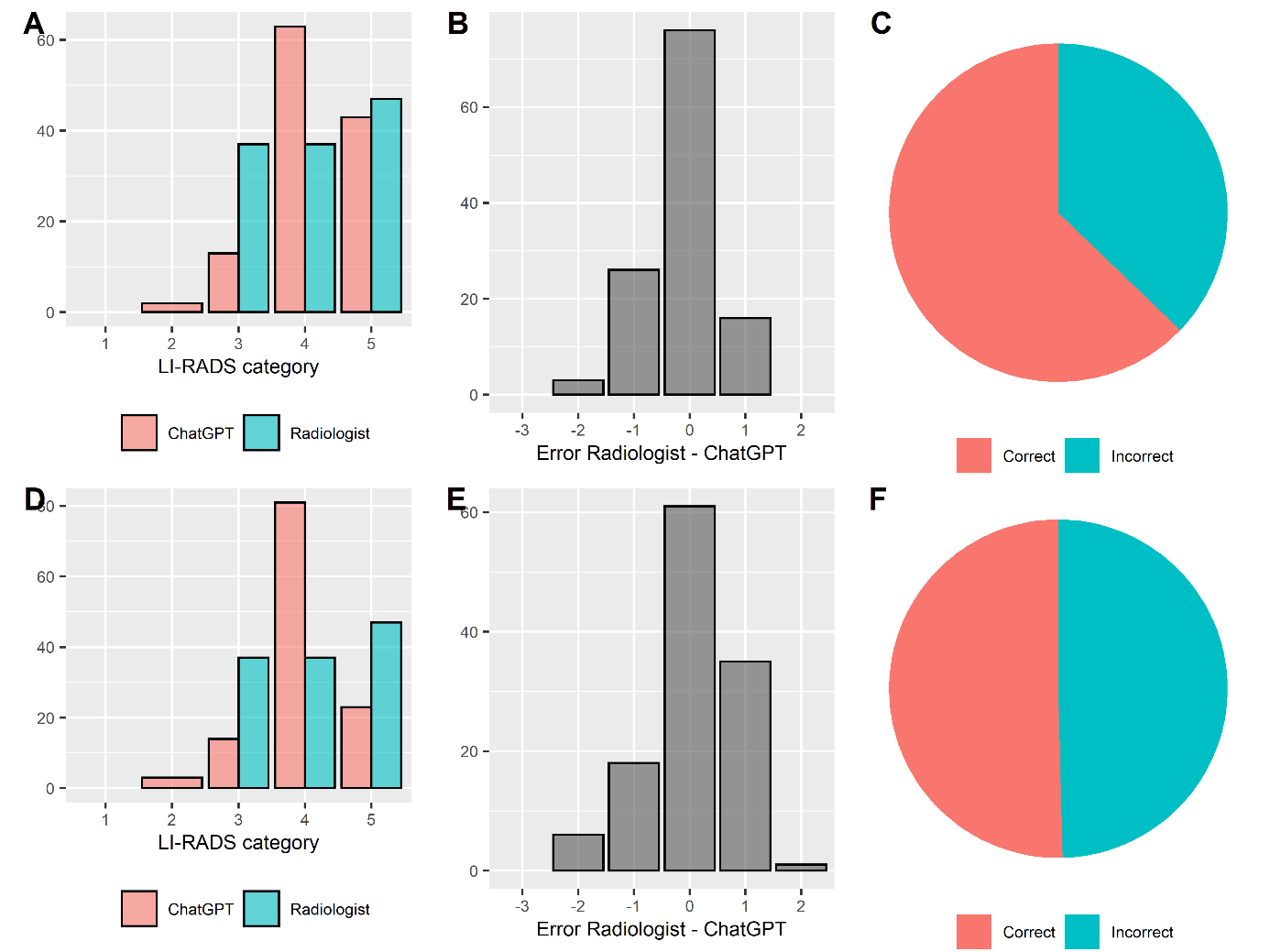


Figure 1: LI-RADS classification performance of ChatGPT based on unstructured and structured radiology reports (balanced dataset).
Performance overview of unstructured and structured reports is shown in the top (A-C) and bottom row (D-F), respectively. (A/D) distribution of the LI-RADS scores, the ground truth (turquoise) scores are distributed equally, (B/E) errors between the experienced liver radiologist and ChatGPT, (C/F) percentage of correct and incorrect LI-RADS classifications by ChatGPT.

Analogously to the analysis in the main manuscript, ChatGPT’s accuracy was superior in unstructured vs. structured reports (correctly classified lesions 63% [76/121] and 50% [61/121], respectively). The Chi square test similarly did not yield statistical significancy (p =0.07).
